# Appraisal of growth inhibitory, biochemical and genotoxic effects of Allyl Isothiocyanate on different developmental stages of *Zeugodacus cucurbitae* (Coquillett) (Diptera: Tephritidae)

**DOI:** 10.1038/s41598-022-14593-0

**Published:** 2022-06-20

**Authors:** Sumit Singh, Diksha, Evani Mahajan, Satwinder Kaur Sohal

**Affiliations:** Department of Zoology, Guru Nanak Dev University, Amritsar, Punjab 143005 India

**Keywords:** Agroecology, Enzymes, Mechanism of action, Screening, Secondary metabolism, Herbivory, Entomology, Physiology

## Abstract

Allyl isothiocyanate (AITC), a glucosinolates’ hydrolytic product, was studied for its anti-insect potential against an economically important, destructive tephritid pest, *Zeugodacus cucurbitae* (Coquillett). The first, second and third instar maggots of the pest were fed on artificial diets amended with varied concentrations of AITC viz. 5 ppm, 25 ppm, 50 ppm, 100 ppm, 150 ppm and 200 ppm with DMSO (0.5%) as control. Results revealed high larval mortality, alteration of larval period, prolongation of pupal and total developmental periods in all instars of the maggots treated with AITC as compared to controls. Percent pupation and percent adult emergence decreased in all larval instars. Growth indices viz. Larval Growth Index (LGI) and Total Growth Index (TGI) were negatively affected. Anti-nutritional/post ingestive toxicity of AITC was also revealed by the decrease in Food Assimilation (FA) and Mean Relative Growth rate (MRGR) values with respect to control. Profiles of PO (Phenol oxidase) and other detoxifying enzymes including SOD (Superoxide dismutases), CAT (Catalases), GST (Glutathione-*S*-transferases), EST (Esterases), AKP (Alkaline phosphatases) and ACP (Acid phosphatases) were also significantly influenced. The genotoxic effect of AITC was also evaluated by conducting comet assays at LC_30_ and LC_50_. Significant DNA damage in hemocytes was reflected by increase in Tail length (μm), Percent Tail DNA, Tail Moment (TM) and Olive Tail Moment (OTM) as compared to controls. The results indicated high potential of AITC as biopesticide for pest management.

## Introduction

As plants are sessile in nature, these cannot run away if they are being approached or attacked by insects, snails or other herbivores. However, in order to avert, restrict or even kill their potential enemies, they synthesise an enormous diversity of organic compounds called as secondary metabolites (SMs)^1,2^. These include glucosinolates, quinines, cyanogenic glycosides, alkaloids, terpenes, saponins etc. and are mainly evolved as the forefront line of defense, but some others also act as olfactory cues for attraction of pollinators^3^. Of these, classical examples that affect insect-plant interactions include glucosinolates (GLTs). GLTs are also known as mustard oil glucosides or S-glucopyranosyl thiohydroximates or (Z)-(or cis)-N-hydroximinosulfate esters. So far, about 120 GLTs have been identified and well illustrated^4,5^. GLTs are usually found to exist in the order Capparales and are particularly abundant in the Brassicaceae family^4,5^.

Plants that accumulate GLTs always have an endogenously present thioglucoside glucohydrolase enzyme (EC 3.2.3.1) known as myrosinase (trivial name)^6^. In intact plants, GLTs and myrosinases are spatially separated but impairment of cellular integrity due to wounding, insect or pathogen attack, mixes them together and the binary GLT-myrosinase system known as ‘mustard oil bomb’ gets stimulated followed by hydrolysis of GLTs leading to the formation of a number of compounds like epithionitrile, oxozolidine-2-thiones, nitriles, thiocyanates and isothiocyanates with diverse biological activities^6^. A majority of the biological functions of GLTs like absolute toxicity, inhibition of development or growth and feeding deterrence activity etc. against a wide variety of herbivores such as birds, slugs and generalist insects are reportedly attributed to the actions of their derived hydrolysis products^7,8^. Among the various hydrolytic products, isothiocyanates (ITCs) have been documented to play a vital role in plant-pathogen interactions^9^. As these plant SMs or botanicals undergo biodegradation within a few hours of exposure^10^, a lot of research is underway to explore these botanical hydrolytic products especially ITCs against various insect pests so as to find compatible and ecologically safer alternatives to harmful synthetic pesticides. Vig et al*.* also reviewed such hydrolytic products as safer biofumigants in pest management studies as these are fully biodegradable and less toxic^4^.

**Figure 1:**
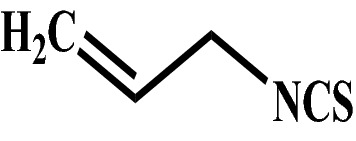
Chemical structure of Allyl isothiocyanate.

ITCs can act as poisons or deterrents against bacteria^11^, fungi^12^ and herbivores^13,14^. Allyl isothiocyanate (AITC) (Fig. 1) is used as an insecticide, bactericide, and nematicide possessing four active EPA registrations in the USA^15^. AITC has been reported to inhibit survival and growth of cabbage white fly, *Pieris rapae* (L.), a specialist lepidopteran insect pest^13^. It has also been reported that AITC generated suppressive effect on the reproductive cycles, particularly on larval and egg stages of two insect pests namely cigarette beetle, *Lasioderma serricorne* (Fabricius) and confused flour beetle, *Tribolium confusum* (Du Val)^16^. Insecticidal efficacy of AITC, extracted from *Armoracia rusticana*, has also been reported against four pests namely mason beetle *Tribolium ferrugineum* (F.), maize weevil *Sitophilus zeamais* (Motsch.), book louse *Liposcelis entomophila* (Enderlein) and lesser grain borer *Rhizopertha dominica* (F.)^17^. Bhushan et al*.* reported insecticidal efficacy of AITC against an insect pest, *Spodoptera litura* (Fabricius)^18^.

*Zeugodacus cucurbitae* (Coquillett) (earlier known by the name *Bactrocera cucurbitae*), commonly called as melon fruit fly, is one of the important agricultural pests that infests and damages a number of fruits and vegetables worldwide^19^. As fruitflies also have quarantine significance, trade is also adversely affected if fruitfly infestation occurs^20^. Native to the Indo-Malayan region, it is found in Burma, Kenya, Southern China, Malaya, Hawaii, India, Mauritius Island, Singapore, Philippines and Timor Island. Melon fruitflies have about 81 hosts including beans, cucumbers, melons, squashes, pumpkins, tomatoes, etc. and the common and usual hosts of melon fruit flies belong to Cucurbitacea family^19^. Voraciously feeding maggots happen to be the most damaging and cause serious damage to the infested crop wherever they occur causing serious economic loss^21^. Vegetation loss due to fruit fly infestation and damage ranges between 30 and100% and particularly in India, this pest has been reported to cause over 40–60% losses in vegetation^19,22^.

ITCs being GLTs hydrolytic products have already been reported to show biofumigant activity against a number of insect pests, especially stored grain pests, but there are no reports showing its post-ingestive insecticidal potency against the destructive tephritid pest, *Z. cucurbitae.* So, the present study aimed at ascertaining the effect of AITC on growth and developmental parameters of *Z. cucurbitae.* Moreover, the genotoxic effect of AITC on the insect pest was also evaluated to ascertain DNA damage induced by AITC in the hemocytes of the pest. Phenol Oxidase (PO), an important enzyme involved in insects’ immune response along with some other detoxifying enzymes were also analyzed for their activity against AITC treatment.

## Results

### Insect bioassays

A significant inhibitory effect of AITC was observed on the various growth and developmental parameters of *Z. cucurbitae*. The percent pupation showed a significant concentration dependent decline (p ≤ 0.01) in all the three larval instars of *Z. cucurbitae* when fed with AITC amended artificial diet (Table 1). At 150 ppm, the decline in percent pupation was maximum in second instar maggots where it decreased by 75.91% as compared to controls. Complete larval mortality was observed at 200 ppm in all the three larval instars. The LC_50_ concentration was lowest for second instar maggots (82.88 ppm) of *Z. cucurbitae*, and was 93.61 ppm for first and 104.36 ppm for third instar maggots.

**Table 1:** Percent Pupation in different larval instars of *Z. cucurbitae* fed on AITC amended artificial diet.

Concentration (ppm)	Pupation (%)		
	First instar	Second instar	Third instar
0	93.33 ± 2.98^a^	92.22 ± 2.05^a^	93.33 ± 2.43^a^
5	84.45 ± 2.81^ab^	81.11 ± 3.18^ab^	88.89 ± 1.4^ab^
25	71.11 ± 5.07^b^	70.00 ± 2.85^b^	80.00 ± 2.44^bc^
50	69.00 ± 4.79^b^	57.78 ± 3.30^c^	72.22 ± 2.05^c^
100	51.11 ± 3.72^c^	47.78 ± 2.05^c^	56.67 ± 2.28^d^
150	32.22 ± 2.05^d^	22.22 ± 2.05^d^	42.22 ± 2.22^e^
F-value	35.744**	88.853**	82.089**

Figures are Mean ± Standard Error. **Significant at 1% level of significance. Means followed by different superscript letters within a column are significantly different. Tukey’s test p ≤ 0.05.

The adult emergence too reduced significantly in all larval instars with increase in concentration of AITC. At highest concentration of 150 ppm, maximum reduction in adult emergence when compared to control was recorded in first larval instars (86.44%) (p ≤ 0.01) (Table 2).

**Table 2:** Adult emergence (%) of different larval instars of *Z. cucurbitae* fed on AITC amended artificial diet.

Concentration (ppm)	Adult emergence (%)		
	First instar	Second instar	Third instar
0	65.56 ± 2.05^a^	66.67 ± 1.72^a^	68.89 ± 2.22^a^
5	49.99 ± 2.28^b^	53.34 ± 2.98^b^	67.78 ± 2.68^a^
25	39.99 ± 4.55^bc^	36.67 ± 1.49^c^	52.22 ± 2.05^b^
50	31.11 ± 4.77^c^	25.56 ± 2.05^d^	44.45 ± 2.22^b^
100	12.00 ± 2.04^d^	18.89 ± 3.62^de^	27.78 ± 2.05^c^
150	8.89 ± 1.40^d^	11.67 ± 1.05^e^	21.11 ± 2.05^c^
F-value	49.039**	82.555**	80.392**

Figures are Mean ± Standard Error. **Significant at 1% level of significance. Means followed by different superscript letters within a column are significantly different. Tukey’s test (p ≤ 0.05).

The observations for larval period also showed significant inhibitory effect of AITC treatment (Table 3). Compared to control, the larval period of first instar maggots shortened significantly with increase in concentration while that of second and third instar maggots prolonged significantly upto 50 ppm AITC concentration, and thereafter, was found to decrease. At highest AITC concentration, there was a shortening of larval period by 0.35 days and 0.19 days in the second and third instar *Z. cucurbitae* maggots, respectively when compared to control.

**Table 3:** Larval period (days) and Pupal period (days) of different larval instars of *Z. cucurbitae* fed on AITC amended artificial diet.

Concentration (ppm)	Larval period (days)			Pupal period (days)		
	First instar	Second instar	Third instar	First instar	Second instar	Third instar
0	9.44 ± 0.45^a^	7.78 ± 0.15^ab^	5.33 ± 0.17^a^	8.89 ± 0.26^a^	9.30 ± 0.24^a^	7.84 ± 0.16^a^
5	8.87 ± 0.36^ab^	8.05 ± 0.07^ab^	5.37 ± 0.22^a^	9.88 ± 0.40^ab^	9.39 ± 0.15^a^	7.84 ± 0.14^a^
25	8.83 ± 0.44^ab^	8.37 ± 0.14^bc^	6.24 ± 0.10^b^	9.74 ± 0.54^ab^	9.89 ± 0.42^a^	8.22 ± 0.10^ab^
50	8.18 ± 0.69^ab^	9.06 ± 0.24^c^	6.30 ± 0.31^b^	10.57 ± 0.74^ab^	9.91 ± 0.27^a^	8.64 ± 0.18^b^
100	7.49 ± 0.29^ab^	7.59 ± 0.24^ab^	5.21 ± 0.15^a^	11.67 ± 0.33^bc^	11.50 ± 0.54^b^	9.96 ± 0.19^c^
150	7.7 ± 0.15^b^	7.43 ± 0.26^a^	5.14 ± 0.11^a^	12.97 ± 0.32^c^	12.82 ± 0.08^b^	10.98 ± 0.28^d^
F-value	3.105*	8.947**	7.608**	10.296**	18.679**	49.404**

Figures are Mean ± Standard Error. *Significant at 5% level of significance. **Significant at 1% level of significance. Means followed by different superscript letters within a column are significantly different. Tukey’s test p ≤ 0.05.

On the other hand, significant prolongation in pupal period and total development period was observed in all the three larval stages of *Z. cucurbitae* after the ingestion of AITC treated diet (p ≤ 0.01) (Tables 3 and 4).

**Table 4:** Total development period (days) of different larval instars of *Z. cucurbitae* fed on AITC amended artificial diet.

Concentration (ppm)	Total development period (days)		
	First instar	Second instar	Third instar
0	18.32 ± 0.28^a^	17.08 ± 0.29^a^	13.17 ± 0.13^a^
5	18.75 ± 0.53^a^	17.45 ± 0.12^ab^	13.21 ± 0.20^a^
25	18.56 ± 0.37^a^	18.26 ± 0.40^bc^	14.46 ± 0.12^b^
50	18.75 ± 0.35^a^	18.97 ± 0.10^c^	14.93 ± 0.21^b^
100	19.15 ± 0.22^a^	19.08 ± 0.30^ cd^	15.17 ± 0.11^b^
150	20.67 ± 0.25^b^	20.25 ± 0.30^d^	16.11 ± 0.24^c^
F-value	5.857**	18.399**	43.8**

Figures are Mean ± Standard Error. **Significant at 1% level of significance. Means followed by different superscript letters within a column are significantly different. Tukey’s test p ≤ 0.05.

After ingestion of AITC amended artificial diet, LGI and TGI was reduced in all the three larval instars of *Z. cucurbitae* (Table 5)*.* Maximum reduction in the LGI was noticed in second instar maggots at highest concentration where it declined by about 74.73% as compared to control (p ≤ 0.01). On the other hand, maximum reduction in the TGI was observed in first instar larvae (87.99%) when compared to control.

**Table 5:** Larval growth index (LGI) and Total growth index (TGI) of different larval instars of *Z. cucurbitae* fed on AITC amended artificial diet.

Concentration (ppm)	Larval growth index (LGI)			Total growth index (TGI)		
	First instar	Second instar	Third instar	First instar	Second instar	Third instar
0	9.95 ± 0.32^a^	11.87 ± 0.28^a^	17.63 ± 0.87^a^	3.58 ± 0.13^a^	3.90 ± 0.09^a^	5.24 ± 0.19^a^
5	9.60 ± 0.49^a^	10.09 ± 0.46^b^	16.68 ± 0.71^a^	2.67 ± 0.14^b^	3.06 ± 0.17^b^	5.14 ± 0.21^a^
25	8.14 ± 0.70^ab^	8.38 ± 0.41^c^	12.86 ± 0.47^b^	2.15 ± 0.23^bc^	2.00 ± 0.05^c^	3.61 ± 0.14^b^
50	8.79 ± 0.78^ab^	6.40 ± 0.42^d^	11.59 ± 0.59^b^	1.68 ± 0.28^c^	1.35 ± 0.10^d^	2.98 ± 0.16^b^
100	6.89 ± 0.59^b^	6.32 ± 0.29^d^	10.95 ± 0.64^c^	0.63 ± 0.11^d^	0.98 ± 0.18^de^	1.83 ± 0.14^c^
150	4.18 ± 0.25^c^	3.00 ± 0.31^e^	8.25 ± 0.47^c^	0.43 ± 0.07^d^	0.58 ± 0.05^e^	1.32 ± 0.14^c^
F-value	14.775**	72.846**	31.09**	46.300**	117.402**	96.571**

Figures are Mean ± Standard Error. **Significant at 1% level of significance. Means followed by different superscript letters within a column are significantly different. Tukey’s test p ≤ 0.05.

**Figure 2:**
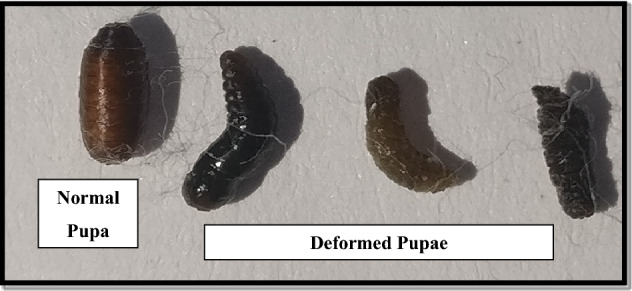
Pupal deformities in *Z. cucurbitae* after feeding maggots on AITC amended artificial diet.

**Figure 3:**
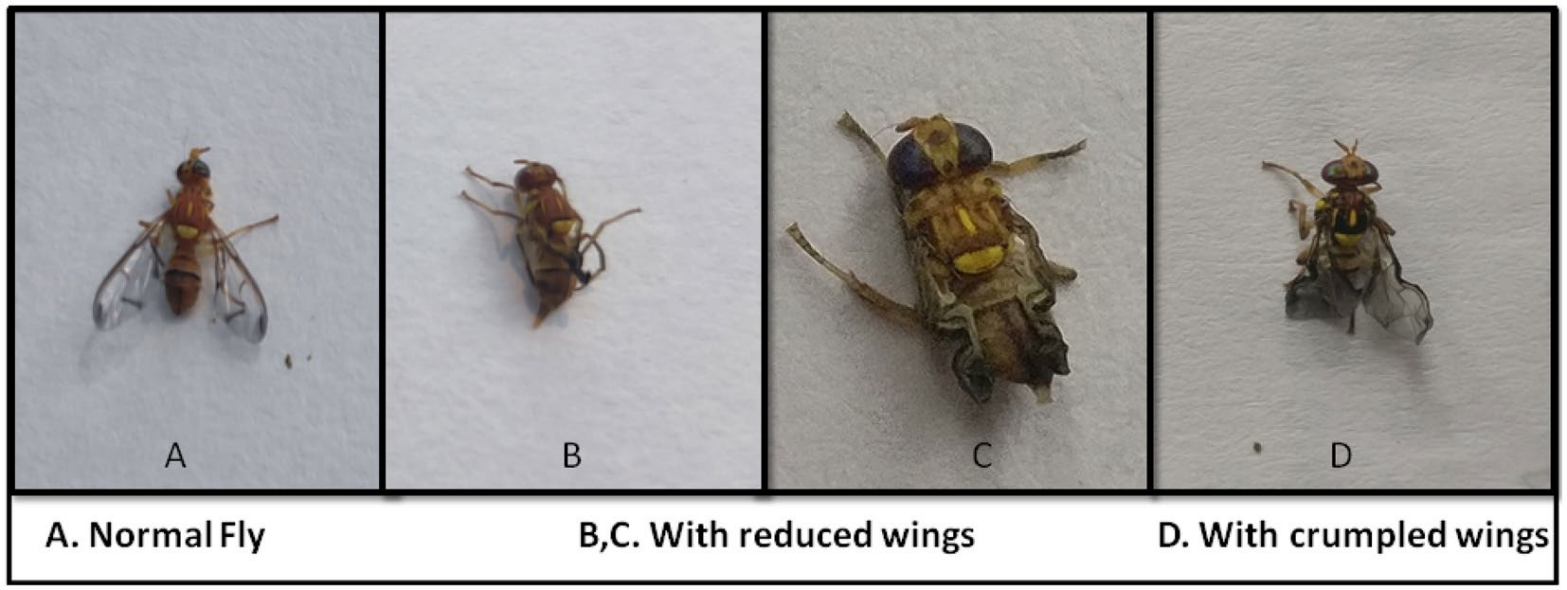
Adult deformities in *Z. cucurbitae* after feeding maggots on AITC amended artificial diet.

Pupal and adult abnormalities were also observed in *Z. cucurbitae* after the maggots were fed on AITC amended artificial diet. Adult abnormalities included distorted abdomen, reduced wings or crumpled wings (Figs. 2, 3).

### Nutritional assay

FA with respect to control decreased significantly from 20.26 mg at 5 ppm to 16.79 mg at 150 ppm (p ≤ 0.01) (Table 6). Similar declining trend was observed in MRGR with maximum decrease at highest concentration (p ≤ 0.01). Larval weight gain (mg) per maggot decreased from 10.48 mg in control upto 6.62 mg at the highest concentration (p ≤ 0.01) (Table 6).

**Table 6:** Nutritional parameters of second instar maggots of *Z. cucurbitae* fed on AITC amended artificial diet.

Concentration (ppm)	FA w.r.t. control (mg)	MRGR (mg/mg/days)	Larval weight gain (mg)
0		0.54 ± 0.005^a^	10.48 ± 0.04^a^
5	20.26 ± 0.22^a^	0.51 ± 0.002^b^	9.71 ± 0.11^b^
25	19.47 ± 0.16^b^	0.48 ± 0.003^c^	8.81 ± 0.10^c^
50	18.93 ± 0.10^b^	0.47 ± 0.001^c^	8.58 ± 0.03^c^
100	17.82 ± 0.16^c^	0.43 ± 0.005^d^	7.31 ± 0.14^d^
150	16.79 ± 0.09^d^	0.41 ± 0.003^e^	6.62 ± 0.03^e^
F-value	78.61**	187.06**	274.35**

FA= Food Assimilated with respect to control, MRGR= Mean relative Growth Rate. Figures are Mean ± Standard error. **Significant at 1% level of significance. Means followed by different superscript letters within a column are significantly different. Tukey’s test p ≤ 0.05.

### Immune and detoxifying enzymes

**Figure 4:**
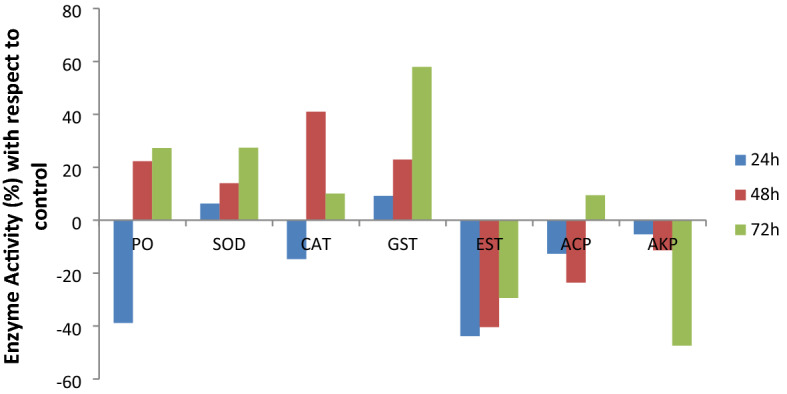
PO (Phenol oxidase), SOD (Superoxide dismutases), CAT (Catalases), GST (Glutathione-*S*-transferases) ACP (Acid phosphatases), AKP (Alkaline phosphatases) and EST (Esterases) activity (%) with respect to control in *Z. cucurbitae* maggots after ingestion of AITC amended artificial diet.

The activity of PO, which plays a vital role in insect immunity, after 24 h of maggots feeding on AITC amended diet showed 38.83% inhibition as compared to control (p ≤ 0.01) but thereafter, the activity was significantly induced in 48 h and 72 h treatment intervals as compared to control (p ≤ 0.01) (Fig. 4).

The activity of antioxidant enzyme, SOD increased with increase in AITC exposure time with maximum increase (27.37%) observed after 72 h of AITC exposure as compared to control (p ≤ 0.01) (Fig. 4). CAT activity too increased after prolonged feeding of the maggots on AITC incorporated diet. At 48 h treatment interval, a 40.97% increase in CAT activity was recorded as compared to control (Fig. 4).

The activity of the detoxification enzyme, GST was significantly induced after consumption of AITC (p ≤ 0.01) (Fig. 4). With respect to control, its activity increased by 57.92% after 72 h treatment interval. On the other hand, the activity of EST was suppressed following AITC treatment of *Z. cucurbitae* maggots (Fig. 4). The suppression in enzyme activity was maximum after 24 h interval (43.79%) and was minimum at 72 h treatment interval (29.41%) with respect to controls (p ≤ 0.01).

The observations made for ACP revealed a decrease in ACP activity at 24 h and 48 h but at 72 h, a 9.45% increase in enzyme activity was observed as compared to control (p ≤ 0.01) (Fig. 4). AKP activity declined in a steady manner at all treatment intervals as compared to control groups (p ≤ 0.01) (Fig. 4).

### Comet assay

**Figure 5:**
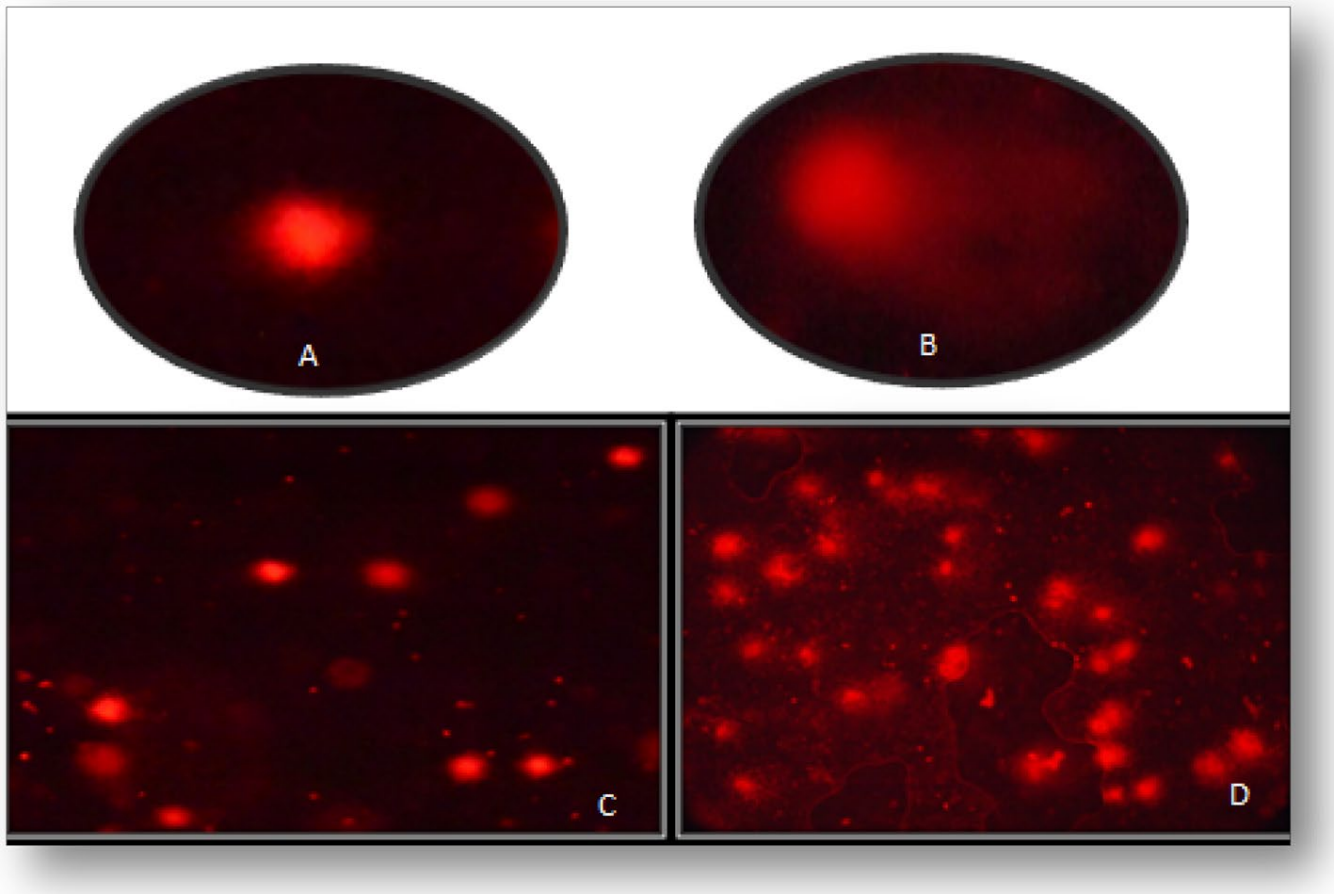
DNA damage in hemocytes of second instar maggots of *Z. cucurbitae* fed on AITC amended artificial diet. (**A**, **C**) Control Maggots; (**B**, **D**) Treated Maggots.

AITC induced DNA damage was also investigated in hemocytes of *Z. cucurbitae* maggots using comet assay (Fig. 5).

The tail length (TL) was significantly increased in a concentration dependent manner in all the three treatment intervals (24 h, 48 h and 72 h) as compared to control but the increase in tail length was observed to be lesser in 48 h treatment groups at LC_50_ concentration as compared to 24 h and 48 h exposure groups (Table 7). After 24 h treatment interval, it significantly increased from 10.45 μm in control to 25.25 μm and 41.50 μm in LC_30_ and LC_50_ concentrations of AITC, respectively (F = 2540.47, p ≤ 0.05). Similar trend was observed in 48 h (F = 405.07, p ≤ 0.05) and 72 h (F = 2270.06, p ≤ 0.05) exposure groups, however, the increase in tail length after 72 h treatment interval was maximum with a value of 42.47 μm in LC_50_ treatment. Similar to tail length, the percent tail DNA values also increased significantly in both the treatment groups (LC_30_ and LC_50_) and at all the treatment intervals as compared to control (F_24h_ = 265.85, p ≤ 0.05; F_48h_ = 360.15, p ≤ 0.05; F_72h_ = 270.77, p ≤ 0.05) (Table 7). In comparison to control, maximum value of 6.2% increase in percent tail DNA was observed in 72 h treatment interval.

**Table 7:** Tail length (TL) (μm) and Percent Tail DNA in hemocytes of second instar maggots of *Z. cucurbitae* fed on AITC amended artificial diet.

Concentration	Tail length (TL) (μm)			%Tail DNA		
	24 h	48 h	72 h	24 h	48 h	72 h
Control	10.45 ± 0.05^a^	9.99 ± 0.08 ^a^	10.27 ± 0.12^a^	2.17 ± 0.12^a^	2.00 ± 0.04^a^	1.37 ± 0.11^a^
LC_30_	25.25 ± 0.35^b^	26.81 ± 0.94^b^	30.33 ± 0.38^b^	4.07 ± 0.27^b^	3.74 ± 0.12^b^	5.84 ± 0.30^b^
LC_50_	41.50 ± 0.40^c^	34.22 ± 0.50^c^	42.47 ± 0.44^c^	7.91 ± 0.10^c^	5.86 ± 0.13^c^	7.58 ± 0.12^c^

Figures are Mean ± Standard Error. Means followed by different superscript letters within a column are significantly different. Tukey’s test p ≤ 0.05.

Likewise, TM and OTM values were analyzed to be significantly and maximally increased in LC_50_ treatment groups after 72 h exposure to AITC amended artificial diet in comparison to control groups (F_TM_ = 2214.97, p ≤ 0.05; F_OTM_ = 1507.35, p ≤ 0.05) (Table 8). The TM and OTM values for LC_50_ fed 72 h treatment groups were 3.33 and 5.93, respectively.

**Table 8:** Tail moment (TM) and Olive tail moment (OTM) in hemocytes of second instar maggots of *Z. cucurbitae* fed on AITC amended artificial diet.

Concentration	Tail moment (TM)			Olive tail moment (OTM)		
	24 h	48 h	72 h	24 h	48 h	72 h
Control	0.26 ± 0.01^a^	0.27 ± 0.01^a^	0.19 ± 0.01^a^	0.92 ± 0.08^a^	0.75 ± 0.01^a^	0.73 ± 0.02^a^
LC_30_	1.17 ± 0.06^b^	1.18 ± 0.08^b^	1.83 ± 0.05^b^	2.73 ± 0.12^b^	2.57 ± 0.03^b^	3.53 ± 0.08^b^
LC_50_	3.64 ± 0.10^c^	2.31 ± 0.07^c^	3.33 ± 0.03^c^	5.82 ± 0.24^c^	4.08 ± 0.02^c^	5.93 ± 0.78^c^

Figures are Mean ± Standard Error. Means followed by different superscript letters within a column are significantly different. Tukey’s test p ≤ 0.05.

## Discussion

### Bioassays

AITC, the hydrolysis product of aliphatic glucosinolates, has been reported to be toxic to a variety of organisms^4,23^. In our study, the exposure of AITC amended diet exhibited dose-dependent larval mortality resulting in a decline in percent pupation in all the larval stages of *Z. cucurbitae*. Although all the larval instars were lethally affected but the effect on early instars (first and second instar maggots) was greater than the third instar larval forms. Ingestive toxicity of GLTs containing extracts has previously been reported against larval forms of mosquito, *Aedes aegypti* (L.) (Diptera: Culicidae) and housefly, *Musca Domestica* (L.) (Diptera: Muscidae)^24^. Bhushan et al*.* also reported insecticidal efficacy of AITC amended artificial diet against larval stages of lepidopteran insect pest, *Spodoptera litura* (Fab.)^18^. Li et al.^25^ too reported insecticidal potential of AITC amended artificial diet against neonates of diamondback moth, *Plutella xylostella* (L.) (Lepidoptera: Plutellidae) and southern armyworm, *Spodoptera eridania* (Cramer) (Lepidoptera: Noctuidae). Noble et al. exposed masked chafer beetle larvae, *Cyclocephala* spp., to soil amended with *Brassica juncea* L. tissue and reported that there existed a positive correlation between the AITC levels and larval mortality, with a mere 8% *B. juncea* demonstrating complete larval mortality containing mean AITC content of 11.4 mg/l of soil atmosphere^26^. AITC even at lower concentrations has been reported to be toxic to the immature larval stages of maize weevil, *S. zeamais* Motschulsky and cowpea weevil, *Callosobruchus maculates* (F.) (Coleoptera: Chrysomelidae) when applied as a fumigant^27,28^. Freitas et al.^29^ and Jabeen et al.^30^ also reported significant toxic and emergence inhibition effects of AITC on insects. The insecticidal toxicity of AITC has reportedly been attributed to its ability to disrupt the normal functioning of two mitochondrial complexes viz. Complex IV (primary target site) and Complex I (as secondary target site), thereby resulting in dysfunctioning of the tissue^31,32^.

ITCs derived from GLTs have been reported to cause mortality, developmental retardation, and deformities in cotton bollworm, *Helicoverpa armigera* Hübner and in other lepidopteran insects^33,34^. In the present study, the larval period shortened with AITC treatment in first larval instar of *Z. cucurbitae* as compared to control but in the second and third larval instars, it showed prolongation at lower concentrations initially but at higher concentrations again got shortened with respect to controls. The shortening of larval period at higher AITC concentrations might be a defense strategy to avert its post-digestive toxicity. However, an overall delay in total developmental period of all the larval instars, attributed mainly to delayed pupal period, was observed when maggots were raised on AITC amended diet as compared to control groups. Agrawal and Kurashige^13^ too reported that AITC decreased survival and growth, and prolonged development time in a dose-dependent manner in a specialist lepidopteran herbivore *P. rapae* (L.). Similar to our study, Jeschke et al.^35^ also reported that the neonates of cotton bollworm, *H. armigera*, raised on GLTs demonstrated significantly prolonged developmental times for all stages.

The delay in developmental duration and late eclosion (i.e. delayed pupal period) with AITC exposure may also suggest quite a few detrimental inferences for the species fitness. A decline in growth rates (LGI and TGI) upon exposure of GLTs or their hydrolytic products, as in the present study for dipteran and in previous reports for different lepidopteran species^33^, may prove to be disadvantageous in a natural setting due to prolongation in exposure time to parasitoids and predators (Hypothesis: Slow growth-high mortality hypothesis) and also to prolonged time period in competing for the food resources. Moreover, late eclosion also comes with the overall fitness cost of producing lesser generations per season^33,36^.

### Nutritional assays

The decrease in MRGR, FA and Larval weight gain with respect to control in the present study also indicated either anti-feedant activity or post-ingestive toxicity induced by AITC amended diet. The delayed development observed in second and third instar maggots as compared to controls may also be correlated to decline in food assimilation efficiency in AITC treated maggots thereby reflecting the anti-nutritional efficacy of the test compound. The cotton bollworm larvae*, H. armigera*, while feeding on *A. thaliana* leaves demonstrated distressed feeding pattern avoiding the GLT-enriched parts particularly, midvein and the leaf edge confirming the deterrent activity of GLTs and their hydrolysis products^37^.

### Immune and detoxifying enzymes

AITC was observed to have a statistically significant influence on the activity of PO enzyme in *Z. cucurbitae*. The enzyme activity was inhibited during the initial 24 h treatment interval as compared to control. A study by Bai et al.^38^ reported that the supression of PO activity resulted in delayed development in case of dipteran pest, *Bactrocera dorsalis* (Hendel). However, an upregulation in PO activity was observed on prolonged exposure (48 h and 72 h) of *Z. cucurbitae* maggots to AITC. Datta et al.^39^ observed a similar induction in PO activity on prolonged exposure of *S. litura* (Fab.) to *Alpinia galanga* (L.) derived extracts and purified compounds eliciting an immune response. The ingestion of AITC in *Z. cucurbitae* larval gut stimulated the immune response via activation of PO system together with the activation of enzymes functioning upstream to prophenoloxidase (ProPO), for example ProPO-activating proteinase^40^ and the consequent production of melanin may probably be linked to either mechanical or proteolytic damage of the epithelium of the gut rather endocytosis^41^. Activation of PO followed by melanisation process are related to production of toxic ROS, subsequent oxidative stress, and increased expenditure of energy, probably explaining the detrimental effects on developmental traits observed in *Z. cucurbitae*. Under natural conditions, these effects might compromise the survival fitness of the population by increasing the susceptibility of the pest to pathogens or other abiotic stressors^41^.

SODs play a key role in the antioxidant defense mechanism of insects and convert toxic O_2_^−^ (superoxide anion) into O_2_ (oxygen) and H_2_O_2_ (hydrogen peroxide)^42,43^. In *Z. cucurbitae* maggots, the activity of SOD increased with increase in exposure time to AITC amended diet as compared to control. In accordance with our findings, Zhang et al.^44^ also reported increased SOD expression following AITC exposure in *S. zeamais*.

In insects, in order to prevent H_2_O_2_ generated via activity of SOD, from being transformed into a highly reactive -OH radical, CAT detoxifies this H_2_O_2_ into H_2_O and O_2_^45^ thereby protecting an insect from detrimental effects of ROS that can attack and degrade various key biomolecules causing a functional decline^46^. In our study, CAT activity initially declined after 24 h AITC treatment compared to control but thereafter increased in 48 h treatment groups as compared to control. Wu et al.^31^ reported similar variations in the CAT activity of AITC exposed adult maize weevil, *S. zeamais*. Zhang et al.^44^ also reported decreased gene expression levels of CAT in *S. zeamais* in response to AITC fumigation (upto 8 h).

ITCs are usually detoxified via conjugation to the nucleophilic thiol (-SH) group of GSH (reduced glutathione) mediated by GSTs as reported in molluscs and generalist lepidopterans such as *H. armigera*,* S. exigua*,* S. littoralis*, cabbage moth, *Mamestra brassicae* (Linnaeus) and cabbage looper, *Trichoplusia ni* (Hübner)^47,48^. Interestingly, this pathway was also reported in dipteran *Scaptomyza* species larvae that are specialist leaf-miners on GLTs rich plants and do not utilize any extracellular biochemical mechanism to prevent formation of ITC, and instead intracellularly metabolize these compounds after being exposed^49^. In the present study, an induction in GST activity after consumption of AITC suggests its intracellular metabolization to some extent. Similar to our findings, GST activity was induced with AITC in adult *S. zeamais*^31^. AITC can irreversibly react with the protein and nucleic acid disulfide and thiol groups thereby causing inactivation of amine metabolic enzymes^31^.

The ESTs are vital detoxification enzymes protecting insects from toxic xenobiotics^50^. EST inhibiting activities, similar to our study, were reported for three ITCs including AITC in red imported fire ant, *Solenopsis invicta* Buren workers^23^. Similarly, ACP activity in the present study was significantly decreased with AITC at 24 h and 48 h treatment intervals and thereafter it increased in 72 h exposure group as compared to controls. AKP activity also declined in a steady manner at all treatment intervals as compared to control groups. Murfadunnisa et al.^51^ reported that the sub-lethal dosage of phytochemicals from *Sphaeranthus amaranthoides* (Sa-EO) suppressed both ACP and AKP activities in *S. litura* larvae. ACP suppression or downregulation with AITC treatment suggests that the phytochemical had reduced the release of phosphorous for energy metabolism, probably resulting in reduced rate of metabolism^52^.

### Comet assays

The comet assay has recently been adapted to assess the levels of genotoxicity or DNA damage in a number of insects’ orders including Orthoptera, Diptera, Lepidoptera and Coleoptera^53^. In the present study, a statistically significant enhancement in all the comet parameters viz. TL (μm), Percent Tail DNA, TM and OTM was recorded in a dose dependent manner, thereby, advocating that the ingestion of AITC amended artificial diet caused significant DNA damage in the hemocytes of second instar maggots of the pest. Single strand breaks occur as a result of DNA damage thereby leading to cell apoptosis^54^ and the formation or lengthening of tail is an indicator of the process of apoptosis as the cells undergoing apoptosis display nuclear fragmentation/disintegration in the form of DNA tails^55^. Insect hemocytes are crucial in providing defensive functions during the growth period, and as such genotoxic damage to hemocytes might suppress the growth and developmental process^56^. Similar to our study, evaluation of genotoxicity of botanicals from *A. galanga* against *S. litura* also showed similar statistically significant increase in TL, percent tail DNA and OTM^57^. IGRs viz. pyriproxyfen and novaluron treated peach fruit fly, *Bactrocera zonata* (Saunders) (Diptera: Tephritidae) also exhibited a significant increase in DNA damage values of TL, % tail DNA and TM in the body cells as compared to control groups^58^.

## Materials and methods

### Flies

*Zeugodacus cucurbitae* culture was originally harvested from the fruit fly infested vegetables, pumpkins and bittergourds, taken from the vegetable markets and then maintained for many generations in the insect culture room of the Insect Physiology Division of the Department of Zoology, Guru Nanak Dev University, Amritsar, Punjab, India. A total of 7–8 insect rearing cages, each one carrying about 300–450 adult flies (males as well as females), were maintained. Every day, fresh pumpkin slices, 15–25% sugar/honey solution and proteinX were given as feed to the adult flies^59^. Aseptic conditions of T (temperature) = 25 ± 3 °C, RH (relative humidity) = 70–80% and PP (Photoperiod) = 10L:14D [light:dark (in h)] meant for the optimum growth of flies were maintained within the insect rearing room.

### Chemical procured

Allyl Isothiocyanate, selected for analyzing its biocidal activity, was purchased from Sigma Aldrich (Product no. 36682).

### Insect bioassays

Insect bioassays were performed on all the three stages of insect maggots viz. first instar (42–48 h old), second instar (66–72 h old) and third instar (88–94 h old) maggots. For experimental purpose, freshly sliced pumpkin pieces were kept inside the rearing cages for about 6–8 h for oviposition by the females. After being charged by the females, these slices were shifted to battery jars and kept inside B.O.D (biological oxygen demand) incubators with maintained T, RH and PP as 25 ± 3 °C, 70–80% and 10L:14D, respectively. The eggs hatch within 24 h. Thereafter, same age maggots were collected by dissecting the pumpkin slices and were released into the experimental vials containing agar based artificial diets^60^ amended with varied concentrations of AITC viz. 5 ppm, 25 ppm, 50 ppm, 100 ppm, 150 ppm and 200 ppm. Stock solution of AITC was prepared in 0.5% DMSO and as such 0.5% DMSO was taken as control. The vials were kept in B.O.D. incubator and observations with respect to growth and developmental parameters like time taken for larval stages to complete, time taken for pupal stage to complete, larval mortality, percent pupation, total development period, pupal and adult deformities were noted after every 24 h. For each concentration, there were 6 replications and a total of 15 maggots were released in each replica.

### Growth indices

The observations made in bioassays were further used for calculating larval growth index (LGI) and total growth index (TGI)^61^.

$${\text{LGI}} = {\text{ Percent }}\;{\text{pupation}}/{\text{Larval }}\;{\text{period}}$$

$${\text{TGI}} = {\text{Percent}}\;{\text{ Emergence}}/{\text{Total}}\;{\text{ Development }}\;{\text{Period}}$$

### Nutritional assay

The nutritional assays were performed using second larval instars only. Maggots were harvested, weighed and transferred to glass vials having artificial diet as in bioassays. For each concentration, there were 6 replications and a total of 15 maggots were released in each replica same as in bioassays. After 48 h of feeding, the maggots were taken out of the vials and weighed again to evaluate change in larval weight. The mean relative growth rate (MRGR)^62^ and Food assimilated (FA)^63^ were calculated as

$$\mathrm{MRGR}\left(\frac{\mathrm{mg}}{\mathrm{mg}}/\mathrm{day}\right)=\frac{\mathrm{logN\,final\,weight } \;\left(\mathrm{mg}\right)-\mathrm{logN\,initial\,weight\,}(\mathrm{mg})}{\mathrm{time }(\mathrm{in\,days})}$$

$$\mathrm{FA }\left(\mathrm{mg}\right)=\mathrm{Ti}\times \frac{\mathrm{Cf}-\mathrm{Ci}}{\mathrm{Ci}}+\mathrm{Tf}-\mathrm{Ti}$$

where, Ci = initial weight of control maggots, Cf = final weight of control maggots, Ti = initial weight of treated maggots, and Tf = final weight of treated maggots.

### Immune and detoxifying enzymes

Enzymatic activities were estimated after 24 h, 48 h and 72 h feeding of second instar maggots on artificial diet amended with LC_50_ concentration of AITC. There were 6 replications for each time interval and a total of 15 maggots were released in each replica same as in bioassays.

#### Phenol oxidase (PO)

PO estimation was done using Zimmer’s methodology^64^. Potassium sodium phosphate buffer (0.05 M, pH 6.2) was used to prepare the larval homogenate as enzyme extract. The assay mixture comprised catechol prepared in buffer (50 mM, pH 6.2) and the enzyme extract. At 340 nm and for 10 min, the increase in absorbance was recorded.

#### Superoxide dismutases (SOD)

The methodology given by Kono with slight modifications was used to estimate SOD activity^65^. Enzyme extract (10% w/v) was prepared in sodium carbonate buffer (50 mM, pH 10.0). The assay mixture contained buffer, enzyme extract, Triton X-100 (0.6%), Nitro-blue tetrazolium (NBT) dye (96 µM) and hydroxylamine hydrochloride (20 mM, pH 6.0). At 540 nm, increase of absorbance was recorded spectrophotometrically for 5 min at 25 °C.

#### Catalases (CAT)

For CAT estimation^66^, 5% (w/v) larval homogenate to be used as enzyme extract was prepared in potassium phosphate buffer (0.05 M, pH 7.0). The assay mixture consisted of enzyme extract and H_2_O_2_ and at 240 nm and 25 °C, the decline in absorbance was recorded for a period of 3 min.

#### Glutathione-*S*-transferases (GST)

The activity of GST was measured using the methodology of Chien and Dauterman^67^. The maggots were homogenized (2% w/v) in sodium phosphate buffer (0.1 M, pH 7.6) containing PTU (0.1 mM). The assay mixture consisted of buffer, GSH solution (50 mM), ethanolic CDNB solution (10 mM in 95% ethanol) and enzyme extract. Spectrophotometrically, at 340 nm and 25 °C, the increase in absorbance was noticed for 5 min.

#### Esterases (EST)

The methodology of Katzenellenbogen and Kafatos^68^ was used to estimate EST activity. Larval homogenate (1% w/v) was prepared in chilled sodium phosphate buffer (0.1 M, pH 6.5). The substrate solution i.e. α-naphthyl acetate (1 mM) in buffer, was incubated for a period of 10 min at a temperature of 30 °C in water bath. Enzyme extract was then added and the tubes were again incubated for 30 min at 30 °C. Then, by adding post coupling solution viz. 5:2 (v/v) Sodium lauryl sulphate (4% w/v) and Fast red TR salt (1% w/v), the reaction was terminated and further incubated for 30 min at 30 °C. At room temperature, absorbance was recorded on spectrophotometer at 540 nm. Standard curve was prepared using serial dilutions of α-naphthol (20–200 µM).

#### Phosphatases: acid phosphatases (ACP) and alkaline phosphatases (AKP)

The estimation of phosphatases was done according to method of Mac Intyre^69^. For ACP, 2% (w/v) larval homogenate was prepared in chilled acetate buffer (0.05 M, pH 5.0). The substrate solution i.e. sodium α-naphthyl phosphate (0.005 M) solution prepared in buffer was pre-incubated for 10 min at 30 °C. The enzyme extract was then added and the resultant mixture was further incubated at a temperature of 30ºC for a period of 30 min. Post-coupling solution consisting of 5:2 (v/v) sodium lauryl sulphate (4% w/v) and Fast red TR salt (0.2% w/v) prepared in buffer was then added to stop the reaction and after one hour, absorbance was measured at 540 nm using spectrophotometer. Using serial dilutions of α-naphthol (0.01 M) standard curve was drawn.

The procedure for the extraction and estimation of AKP was similar to that of ACP except that the Tris buffer (0.05 M, pH 8.6) was used to prepare 1% (w/v) larval homogenate in case of ACP.

### Comet assay

Comet assay also known as Single Cell Gel Electrophoresis^70^ was conducted to evaluate the genotoxic effect of AITC (LC_30_ and LC_50_) on insect hemocytes after 24 h, 48 h and 72 h feeding intervals. The steps include:

#### Preparation of sample

10 maggots were pooled and their heads were clipped off. The hemolymph that oozed out was collected in eppendorfs and immediately mixed with phosphate buffer saline (PBS) (pH 7.4) and used for comet assay. Each sample vial contained 10 μl hemolymph homogenized with 40 μl PBS.

#### Lysis buffer

Initially, 445 ml stock solution of the lysis buffer was prepared (73.01 g NaCl, 18.7 g EDTA, 0.6 g Tris and 4 g NaOH) in dH_2_O (pH 10) and then, DMSO (44.5 ml) and Triton X (4.95 ml) were added to the stock solution in order to prepare a working solution.

#### Buffers: electrophoresis buffer and tris buffer

Again, Stock solutions of components of electrophoresis buffer viz. NaOH (40 g/100 ml ddH_2_O) and EDTA (7.44 g/100 ml ddH_2_O) were prepared separately. Then, 30 ml of NaOH and 5 ml of EDTA were taken and mixed with 965 ml of chilled ddH_2_O to form the working solution. Tris buffer (pH 7.4) comprised of 4.84 g of Tris buffer dissolved in 100 ml ddH_2_O.

#### Preparation of slide and electrophoresis

Base layer of 1% NMPA (Normal Melting Point Agarose) was applied over the glass slide and the slide was kept undisturbed for about 12–24 h. After 12–24 h, the second layer of 35 μl hemolymph sample mixed with 110 μl of 0.5% LMPA (Low Melting Point Agarose) was laid over the slide and the slide kept in refrigerator (4 °C) for about 15 min for gel casting. Finally, the top third layer of 0.5% LMPA was laid and again the slide was kept in refrigerator for the same period as in second layering. The slide was then completely immersed in the working lysis buffer for about 2–3 h at 4 °C in dark. After lysis, the slide was transferred to electrophoresis tank and then, electrophoresis buffer was poured upto a level that the slide was completely dipped in it. Following electrophoresis (300 mA, 20 V, 20 min), the slide was taken out and neutralized using Tris buffer. Finally, the slide was washed using chilled dH_2_O.

#### Staining and analysis

Staining was done using EtBr (ethidium bromide) and the slide was transferred under Fluorescent microscope (Nikon ECLIPSE E200), observed and photographed using camera (Nikon D5300). For each treatment, 150 cells were analysed using Casp Lab Software for Tail length (TL) (μm), Percent Tail DNA, Tail Moment (TM) and Olive Tail Moment (OTM) values to assess the degree of DNA damage.

### Statistical analysis

The statistical analyses viz. ANOVA (One-way analysis of variance) and Tukey’s test for comparison of means (*p* ≤ 0.05) for bioassays, nutritional assays and Comet assays were done using SPSS Statistical software Version no. 16. Data from biochemical work was also analysed using SPSS software by applying student ‘t’ test.

## Conclusion

The results of the present study, viz. larval mortality, growth inhibition, developmental delay, disruption of DNA integrity, alterations in profiles of immune and detoxifying enzymes and the reduced species fitness of the pest *Z. cucurbitae* after AITC treatment, indicated its high potential to be employed as a biopesticide for pest management studies especially for *Z. cucurbitae.* The study also provides baseline data that can be explored by plant breeders to make *Z. cucurbitae* resistant varieties by introducing AITC (or Allyl GLTs) encoding genes in those plants that are usually damaged by this destructive pest and do not form GLTs producing AITC.

## Data Availability

The datasets used and/or analyzed during the current study are available from the corresponding author on reasonable request.

## References

[1] Wink M (2003). Evolution of secondary metabolites from an ecological and molecular phylogenetic perspective. Phytochemistry.

[2] Khare S (2020). Plant secondary metabolites synthesis and their regulations under biotic and abiotic constraints. J. Plant Biol..

[3] Gajger IT, Dar SA (2021). Plant allelochemicals as sources of insecticides. Insects.

[4] Vig AP, Rampal G, Thind TS, Arora S (2009). Bio-protective effects of glucosinolates: A review. LWT Food Sci. Technol..

[5] Sikorska-Zimny K, Beneduce L (2020). The glucosinolates and their bioactive derivatives in *Brassica*: A review on classification, biosynthesis and content in plant tissues, fate during and after processing, effect on the human organism and interaction with the gut microbiota. Crit. Rev. Food Sci. Nutr..

[6] Radojčić Redovniković I, Glivetić T, Delonga K, Vorkapić-Furač J (2008). Glucosinolates and their potential role in plant. Period. Biol..

[7] Wittstock U, Kliebenstein DJ, Lambrix V, Reichelt M, Gershenzon J (2003). Chapter five glucosinolate hydrolysis and its impact on generalist and specialist insect herbivores. Recent Adv. Phytochem..

[8] Noret N (2005). Palatability of *Thlaspi caerulescens* for snails: Influence of zinc and glucosinolates. New Phytol..

[9] Hopkins RJ, Van Dam NM, Van Loon JJA (2008). Role of glucosinolates in Insect-plant relationships and multitrophic interactions. Annu. Rev. Entomol..

[10] Guleria S, Tiku AK (2009). Botanicals in pest management: Current status and future perspectives. Integr. Pest Manag..

[11] Clay NK, Adio AM, Denoux C, Jander G, Ausubel FM (2009). Glucosinolate metabolites required for an *Arabidopsis* innate immune response. Science.

[12] Yang B (2021). Inhibitory effect of allyl and benzyl isothiocyanates on ochratoxin a producing fungi in grape and maize. Food Microbiol..

[13] Agrawal AA, Kurashige NS (2003). A role for isothiocyanates in plant resistance against the specialist herbivore *Pieris rapae*. J. Chem. Ecol..

[14] Müller C (2018). The role of the glucosinolate-myrosinase system in mediating greater resistance of *Barbarea verna* than *B. vulgaris* to *Mamestra brassicae *Larvae. J. Chem. Ecol..

[15] Kegley SE, Hill BR, Orme S, Choi AH (2000). PAN Pesticide Database.

[16] Worfel RC, Schneider KS, Yang TCS (1997). Suppressive effect of allyl isothiocyanate on populations of stored grain insect pests. J. Food Process. Preserv..

[17] Wu H, Zhang GA, Zeng S, Lin KC (2009). Extraction of allyl isothiocyanate from horseradish (*Armoracia rusticana*) and its fumigant insecticidal activity on four stored-product pests of paddy. Pest Manag. Sci..

[18] Bhushan S, Gupta S, Kaur Sohal S, Arora S, Saroj Arora C (2016). Assessment of insecticidal action of 3-Isothiocyanato-1-propene on the growth and development of *Spodoptera litura* (Fab.) (Lepidoptera: Noctuidae). J. Entomol. Zool. Stud..

[19] Dhillon MK, Naresh JS, Singh R, Sharma NK (2005). Reaction of different bitter gourd (*Momordica charantia* L.) genotypes to melon fruit fly, *Bactrocera cucurbitae* (Coquillett). Int. J. Plant Prot..

[20] Ekesi S, Nderitu PW, Chang CL (2007). Adaptation to and small-scale rearing of invasive fruit fly *Bactrocera invadens* (Diptera: Tephritidae) on artificial diet. Ann. Entomol. Soc. Am..

[21] Jakhar S (2020). Estimation losses due to fruit fly, *Bactrocera cucurbitae* (Coquillett) on long melon in semi-arid region of Rajasthan. J. Entomol. Zool. Stud..

[22] Ladania MS (2008). Physiological Disorders and Their Management. Citrus Fruit: Biology, Technology and Evaluation.

[23] Du Y, Grodowitz MJ, Chen J (2020). Insecticidal and enzyme inhibitory activities of isothiocyanates against red imported fire ants, *Solenopsis invicta*. Biomolecules.

[24] Tsao R, Reuber M, Johnson L, Coats JR (1996). Insecticidal toxicities of glucosinolate· containing extracts from crambe seeds. J. Agric. Urban Entomol..

[25] Li Q, Eigenbrode SD, Stringam GR, Thiagarajah MR (2000). Feeding and growth of *Plutella xylostella* and *Spodoptera eridania* on *Brassica juncea* with varying glucosinolate concentrations and myrosinase activities. J. Chem. Ecol..

[26] Noble RR, Harvey SG, Sams CE (2002). Toxicity of Indian mustard and allyl isothiocyanate to masked chafer beetle larvae. Plant Health Prog..

[27] Sousa AH, Faroni LRA, Pimentel MAG, Freitas RS (2014). Relative toxicity of mustard essential oil to insect-pests of stored products. Rev. Caatinga.

[28] de Souza LP, Faroni LRDA, Lopes LM, de Sousa AH, Prates LHF (2018). Toxicity and sublethal effects of allyl isothiocyanate to *Sitophilus zeamais* on population development and walking behavior. J. Pest Sci..

[29] Freitas RCP, Faroni LRDA, Haddi K, Jumbo LOV, Oliveira EE (2016). Allyl isothiocyanate actions on populations of *Sitophilus zeamais* resistant to phosphine: Toxicity, emergence inhibition and repellency. J. Stored Prod. Res..

[30] Jabeen A, Zaitoon A, Lim LT, Scott-Dupree C (2021). Toxicity of five plant volatiles to adult and egg stages of *Drosophila suzukii* matsumura (Diptera: Drosophilidae), the spotted-wing *Drosophila*. J. Agric. Food Chem..

[31] Wu H, Liu XR, Yu DD, Zhang X, Feng JT (2014). Effect of allyl isothiocyanate on ultra-structure and the activities of four enzymes in adult *Sitophilus zeamais*. Pestic. Biochem. Physiol..

[32] Zhang C, Wu H, Zhao Y, Ma Z, Zhang X (2016). Comparative studies on mitochondrial electron transport chain complexes of *Sitophilus zeamais* treated with allyl isothiocyanate and calcium phosphide. Pestic. Biochem. Physiol..

[33] Jeschke V, Kearney EE, Schramm K, Kunert G, Shekhov A, Gershenzon J, Vassão DG (2017). How glucosinolates affect generalist lepidopteran larvae: Growth, development and glucosinolate metabolism. Front Plant Sci..

[34] Agnihotri AR, Hulagabali CV, Adhav AS, Joshi RS (2018). Mechanistic insight in potential dual role of sinigrin against *Helicoverpa armigera*. Phytochemistry.

[35] Jeschke V, Zalucki JM, Raguschke B, Gershenzon J, Heckel DG, Zalucki MP, Vassão DG (2021). So much for glucosinolates: A generalist does survive and develop on Brassicas, but at what cost?. Plants.

[36] Benrey B, Denno RF (1997). The slow-growth-high-mortality hypothesis: A test using the cabbage butterfly. Ecology.

[37] Shroff R, Vergara F, Muck A, Svatoš A, Gershenzon J (2008). Nonuniform distribution of glucosinolates in *Arabidopsis thaliana* leaves has important consequences for plant defense. Proc. Natl. Acad. Sci.

[38] Bai PP, Chen EH, Shen GM, Wei D, Wei DD, Wang JJ (2014). Inhibition of phenoloxidase activity delays development in *Bactrocera dorsalis* (Diptera: Tephritidae). Fla. Entomol..

[39] Datta R, Kaur A, Saraf I, Singh IP, Kaur S (2018). Effect of ethyl acetate extract and purified compounds of *Alpinia galanga* (L.) on Immune Response of a *Polyphagous Lepidopteran *pest, *Spodoptera litura* (Fabricius). Asian J. Adv. Basic Sci..

[40] Hartzer KL, Zhu KY, Baker JE (2005). Phenoloxidase in larvae of *Plodia interpunctella* (Lepidoptera: Pyralidae): Molecular cloning of the proenzyme cDNA and enzyme activity in larvae paralyzed and parasitized by *Habrobracon hebetor* (Hymenoptera: Braconidae). Arch. Insect Biochem. Physiol..

[41] Silva CJM, Beleza S, Campos D, Soares AMVM, Patrício Silva AL, Pestana JLT, Gravato C (2021). Immune response triggered by the ingestion of polyethylene microplastics in the dipteran larvae *Chironomus riparius*. J. Hazard. Mater..

[42] Aucoin Richard R., Philogène Bernard J. R., Arnason John T. (1991). Antioxidant enzymes as biochemical defenses against phototoxin-induced oxidative stress in three species of herbivorous lepidoptera. Archives of Insect Biochemistry and Physiology.

[43] Wang Y, Branicky R, Noë A, Hekimi S (2018). Superoxide dismutases: Dual roles in controlling ROS damage and regulating ROS signaling. Int. J. Cell Biol..

[44] Zhang C, Ma Z, Zhang X, Wu H (2017). Transcriptomic alterations in *Sitophilus zeamais* in response to allyl isothiocyanate fumigation. Pest. Biochem. Physiol..

[45] Felton GW, Summers CB (1995). Antioxidant systems in insects. Arch. Insect Biochem. Physiol..

[46] Cadenas E, Ahmad S (1995). Mechanisms of oxygen activation and reactive oxygen species detoxification. Oxidative Stress and Antioxidant Defenses in Biology.

[47] Schramm K, Vassão DG, Reichelt M, Gershenzon J, Wittstock U (2012). Metabolism of glucosinolate- derived isothiocyanates to glutathione conjugates in generalist lepidopteran herbivores. Insect Biochem. Mol. Biol..

[48] Falk KL (2014). The role of glucosinolates and the jasmonic acid pathway in resistance of *Arabidopsis thaliana* against molluscan herbivores. Mol. Ecol..

[49] Gloss AD (2014). Evolution in an ancient detoxification pathway is coupled with a transition to herbivory in the Drosophilidae. Mol. Biol. Evol..

[50] Bhatt P, Zhou X, Huang Y, Zhang W, Chen S (2021). Characterization of the role of esterases in the biodegradation of organophosphate, carbamate, and pyrethroid pesticides. J. Hazard. Mater..

[51] Murfadunnisa S, Vasantha-Srinivasan P, Ganesan R, Senthil-Nathan S, Kim TJ, Ponsankar A, Krutmuang P (2019). Larvicidal and enzyme inhibition of essential oil from *Spheranthus amaranthroids* (Burm.) against lepidopteran pest *Spodoptera litura *(Fab.) and their impact on non-target earthworms. Biocatal. Agric. Biotechnol..

[52] Sengottayan SN (2013). Physiological and biochemical effect of neem and other Meliaceae plants secondary metabolites against Lepidopteran insects. Front. Physiol..

[53] Augustyniak M, Gladysz M, Dziewięcka M (2016). The Comet assay in insects: Status, prospects and benefits for science. Mutat. Res. Rev. Mutat. Res..

[54] Foster ER, Downs JA (2005). Histone H2A phosphorylation in DNA double strand break repair. FEBS J..

[55] Porichha SK, Sarangi PK, Prasad R (1998). Genotoxic effect of chlorpyrifosin *Channa punctatus*. Cytol. Genet..

[56] Kalita MK, Haloi K, Devi D (2016). Larval exposure to chlorpyrifos affects nutritional physiology and induces genotoxicity in silkworm *Philosamia ricini* (Lepidoptera: Saturniidae). Front. physiol..

[57] Datta R, Kaur A, Saraf I, Kaur M, Singh IP, Chadha P, Kaur S (2020). Assessment of genotoxic and biochemical effects of purified compounds of *Alpinia galanga* on a polyphagous lepidopteran pest *Spodoptera litura* (Fabricius). Phytoparasitica.

[58] Afify A, Negm AAKH (2018). Genotoxic effect of insect growth regulators on different stages of peach fruit fly, *Bactrocera zonata* (Saunders)(Diptera: Tephritidae). Afr. Entomol..

[59] Gupta JN, Verma AN, Kashyap RK (1978). An improved method for mass rearing for melon fruit fly *Dacus cucurbitae* Coquillett. Indian J. Entomol..

[60] Srivastava BG (1975). A chemically defined diet for *Dacus cucurbitae* (Coq.) larvae under aseptic conditions. Entomol. News Lett..

[61] Kumar A, Sood S, Mehta V, Nadda G, Shanker A (2004). Biology of *Thysanoplusia orichalcea* (Fab.) in relation to host preference and suitability for insect culture and bioefficacy. Indian J. Appl. Entomol..

[62] Martinez SS, Emden HFV (2001). Growth disruption, abnormalities and mortality of *Spodoptera littoralis* (Boisduval) (Lepidoptera: Noctuidae) caused by azadirachtin. Neotrop. Entomol..

[63] Khan ZR, Saxena RC (1985). Behavioural and physiological responses of *Sogatella furcifera* (Homoptera: Delphacidae) to selected resistant and susceptible rice cultivars. J. Econ. Entomol..

[64] Zimmer M, Graça MA, Bärlocher F, Gessner MO (2005). Phenol oxidation. Methods to Study Litter Decomposition.

[65] Kono Y (1978). Generation of superoxide radical during auto-oxidation of hydroxylamine and an assay for superoxide dismutase. Arch. Biochem. Biophys..

[66] Bergmeyer HU, Bergmeyer HU, Gawehn K (1974). Reagents for enzymatic analysis. Methods of Enzymatic Analysis.

[67] Chien C, Dauterman WC (1991). Studies on glutathione S-transferases in *Helicoverpa* (*=Heliothis*) *zea*. Insect Biochem..

[68] Katzenellenbogen B, Kafatos FC (1971). General esterases of silk worm moth moulting fluid: Preliminary characterization. J. Insect Physiol..

[69] Mac Intyre RJ (1971). A method for measuring activities of acid phosphatases separated by acrylamide gel electrophoresis. Biochem. Genet..

[70] Singh NP, McCoy MT, Tice RR, Schneider EL (1988). A simple technique for quantitation of low levels of DNA damage in individual cells. Exp. Cell Res..

